# *CYP2D6* Genetic Variation and Its Implication for Vivax Malaria Treatment in Madagascar

**DOI:** 10.3389/fphar.2021.654054

**Published:** 2021-04-20

**Authors:** Rajeev K. Mehlotra, Andrea Gaedigk, Rosalind E. Howes, Tovonahary A. Rakotomanga, Arsene C. Ratsimbasoa, Peter A. Zimmerman

**Affiliations:** ^1^ Center for Global Health and Diseases, Case Western Reserve University School of Medicine, Cleveland, OH, United States; ^2^ Division of Clinical Pharmacology, Toxicology & Therapeutic Innovation, Children’s Mercy Kansas City, Kanas City, MO, United States; ^3^ Big Data Institute, Li Ka Shing Centre for Health Information and Discovery, University of Oxford, Oxford, United Kingdom; ^4^ Foundation for Innovative New Diagnostics, Geneva, Switzerland; ^5^ The National Malaria Control Program, Ministry of Health, Antananarivo, Madagascar; ^6^ University of Fianarantsoa, Fianarantsoa, Madagascar

**Keywords:** CYP2D6, hypnozoite, Madagascar, malaria treatment, *Plasmodium vivax*, primaquine

## Abstract

*Plasmodium vivax* is one of the five human malaria parasite species, which has a wide geographical distribution and can cause severe disease and fatal outcomes. It has the ability to relapse from dormant liver stages (hypnozoites), weeks to months after clearance of the acute blood-stage infection. An 8-aminoquinoline drug primaquine (PQ) can clear the hypnozoites, and thus can be used as an anti-relapse therapeutic agent. Recently, a number of studies have found that its efficacy is compromised by polymorphisms in the cytochrome P450 2D6 (*CYP2D6*) gene; decreased or absence of CYP2D6 activity contributes to PQ therapeutic failure. The present study sought to characterize *CYP2D6* genetic variation in Madagascar, where populations originated from admixture between Asian and African populations, vivax malaria is endemic, and PQ can be deployed soon to achieve national malaria elimination. In a total of 211 samples collected from two health districts, CYP2D6 decreased function alleles CYP2D6*10, *17, *29, *36+*10, and *41 were observed at frequencies of 3.55–17.06%. In addition, nonfunctional alleles were observed, the most common of which were CYP2D6*4 (2.13%), *5 (1.66%), and the *4x2 gene duplication (1.42%). Given these frequencies, 34.6% of the individuals were predicted to be intermediate metabolizers (IM) with an enzyme activity score (AS) ≤ 1.0; both the IM phenotype and AS ≤ 1.0 have been found to be associated with PQ therapeutic failure. Furthermore, the allele and genotype frequency distributions add to the archaeological and genomic evidence of Malagasy populations constituting a unique, Asian-African admixed origin. The results from this exploratory study provide fresh insights about genomic characteristics that could affect the metabolism of PQ into its active state, and may enable optimization of PQ treatment across human genetic diversity, which is critical for achieving *P. vivax* elimination.

## Introduction

Five *Plasmodium* species are known to cause malaria in humans; these are, *Plasmodium falciparum*, *P. vivax*, *P. malariae*, *P. ovale*, and *P. knowlesi*. Although *P. vivax* has often been regarded as causing a benign self-limiting infection, there is clear evidence that it can cause severe disease and fatality (Baird, 2009; Battle et al., 2012; Baird, 2013). It has a wide geographical distribution, with relatively high prevalence of infection in the South-East Asian and Western Pacific regions and in most of the Americas (Howes et al., 2016; Battle et al., 2019). Within Africa, countries around the Horn of Africa and Madagascar, which have a lower prevalence of the human Duffy-negative phenotype, once thought to provide complete protection against *P. vivax* (Miller et al., 1976), can sustain endemic transmission of this parasite (Menard et al., 2010; Howes et al., 2011; Howes et al., 2015; Twohig et al., 2019). There is now substantial evidence that *P. vivax* is endemic throughout Africa (Howes et al., 2015; Twohig et al., 2019), though local surveillance for this parasite remains rare (Zimmerman, 2017).

The challenges in controlling and eliminating vivax malaria are likely to be related to a number of unique aspects of *P. vivax* biology (Adams and Mueller, 2017; Bourgard et al., 2018), mainly its ability to relapse from long-lasting, dormant liver stages (the hypnozoites) (Imwong et al., 2007; White, 2011; White and Imwong, 2012; Battle et al., 2014). The hypnozoites persist in the liver, from weeks to months, and cause relapses after clearance of the acute blood-stage infection (White, 2011; Battle et al., 2014). As there are no diagnostic tests that can identify these dormant liver-stage infections, undetectable hypnozoite carriers are an important potential parasite reservoir for sustaining transmission and reintroduction, if not treated appropriately. From the 1950s until 2018, 8-aminoquinoline primaquine (PQ) was the only licensed drug available to clear the hypnozoites (John et al., 2012; Baird, 2019). In 2018, the United States Food and Drug Administration approved the use of a new hypnozoitocidal 8-aminoquinoline called tafenoquine (Baird, 2018; White, 2019). Although these two 8-aminoquinoline drugs are the only likely near-term anti-relapse therapies currently available, their safety and efficacy is severely limited by host genetic variation: patients with a deficiency in glucose-6-phosphate dehydrogenase (G6PD) enzyme activity levels can have acute hemolytic anemia (Baird et al., 2018; Baird, 2019; White, 2019). In addition, clinical and laboratory evidence suggested that the efficacy of PQ may depend on genetic variation in cytochrome P450 2D6 (CYP2D6) drug-metabolizing enzyme activity (Baird, 2018; Baird et al., 2018; Baird, 2019).

CYP2D6 is involved in the metabolism of up to 21% of drugs, many of which are commonly prescribed (Saravanakumar et al., 2019). Among these is PQ, which has been shown to be metabolized principally via CYP2D6 in animal models (Pybus et al., 2013; Potter et al., 2015), *in vitro* enzymatic assays (Fasinu et al., 2014; Fasinu et al., 2016a; Fasinu et al., 2016b; Saito et al., 2018; Camarda et al., 2019), and in human studies (Goncalves et al., 2017; Ariffin et al., 2019; Spring et al., 2019). CYP2D6 also contributes to the metabolism of tafenoquine (Marcsisin et al., 2014; Vuong et al., 2015). CYP2D6 is among the most extensively studied and characterized polymorphic drug-metabolizing enzymes (Taylor et al., 2020). Variation in the *CYP2D6* gene includes single nucleotide variants, short insertions and deletions, as well as gene copy number variations (CNV). The latter encompass deletions of the entire gene, gene duplications and multiplications, and structural rearrangements with the highly similar *CYP2D7* pseudogene (Nofziger et al., 2020). Currently, there are over 130 *CYP2D6* “star” (*) alleles listed by the Pharmacogene Variation Consortium (PharmVar) (Nofziger et al., 2020). Many of the listed alleles contribute, in part or entirely, to altered rates of CYP2D6-mediated drug metabolism due to increased, decreased, or absent enzyme activity (Nofziger et al., 2020; Taylor et al., 2020). A system for translating *CYP2D6* genotype to phenotype, and assigning individuals into four phenotype groups [ultrarapid metabolizers (UM), normal metabolizers (NM), intermediate metabolizers (IM), and poor metabolizers (PM)], has been developed by the Clinical Pharmacogenetic Implementation Consortium (CPIC) (Caudle et al., 2020).

In a clinical trial of a vaccine against *P. vivax* involving 25 healthy malaria-naïve adults, who were given a combination of chloroquine and PQ as treatment, two participants had multiple relapses of malaria (Bennett et al., 2013). One participant who experienced two relapses was genotyped as CYP2D6*4/*41 and classified as IM, whereas another participant who experienced three relapses was a PM with a CYP2D6*5/*6 genotype*.* In another study, an IM subject (genotyped as CYP2D6*5/*41) suffered multiple attacks of vivax malaria at approximately two-month intervals despite taking PQ as prescribed (Ingram et al., 2014). Since those findings were reported (Bennett et al., 2013; Ingram et al., 2014), there have been several other reports suggesting that decreased or absence of CYP2D6 activity contributes to PQ therapeutic failure (Baird et al., 2018; Dijanic et al., 2018; He et al., 2019; Martin Ramirez et al., 2020; Mat Salleh et al., 2020; Silvino et al., 2020). In contrast, in clinical trials using tafenoquine in combination with chloroquine, the IM phenotype was reported to have had no significant effect on the efficacy of tafenoquine in preventing *P. vivax* relapse; however, more data on PM persons are required (St Jean et al., 2016; Lacerda et al., 2019).

Madagascar is an island nation in the Indian Ocean, approximately 400 km (250 miles) off the East African coast. Archaeological and genomic data provide evidence of sequential waves of human migration over the last two millennia to Madagascar from Indonesia and from East Africa by Bantu populations, together with more limited later arrivals of Arab, European, Indian, and Chinese peoples. These events have resulted in a genetically cosmopolitan Malagasy society (Kusuma et al., 2015; Brucato et al., 2016; Kusuma et al., 2016; Pierron et al., 2017; Pierron et al., 2018). *Plasmodium falciparum* is the predominant malaria species in Madagascar, and *P. vivax*, *P. malariae* and *P. ovale* are also present. *Plasmodium vivax* is diagnosed at similar prevalence as *P. falciparum* by molecular methods in the western highlands fringe region (Howes et al., 2018). Irrespective of the parasite species involved, any malaria positive cases are treated with an age-adjusted dose of artesunate-amodiaquine. *Plasmodium vivax*-specific treatment of hypnozoites in the form of PQ for radical cure is not available, although vivax relapse has been reported (Andrianaranjaka et al., 2013). The Madagascar National Malaria Control (NMCP) Program 2018–2022 has declared its objective for achieving local elimination in low-burden highland districts while gradually pushing back residual transmission to coastal areas, en route to achieving national elimination (National Malaria Control Programme of Madagascar, 2017). Therefore, assessing PQ efficacy in the target *P. vivax* patient population is essential to support the malaria elimination goal. Although *CYP2D6* genetic variation has been extensively studied in many populations, there are no published data for the Malagasy, a rather unique population with a rich admixture history between Asian and African ancestral populations and the potential for novel genotypic combinations.

To reduce this knowledge gap, the aim of the present study was to characterize *CYP2D6* genetic variation in a Malagasy population exposed to *P. vivax* transmission. Given that CYP2D6 emerges as a major pathway for PQ bioactivation, and decreased or absence of enzyme activity may contribute to PQ therapeutic failure, *CYP2D6* genetic variation and its functional implications must be understood to develop strategies and expectations for *P. vivax* elimination in Madagascar as well as other *P. vivax* endemic settings.

## Methods

### Subjects and Study Sites

This investigation is a part of our ongoing malaria epidemiological studies being conducted in the western highlands fringe region of Madagascar (Menard et al., 2010; Howes et al., 2017; Howes et al., 2018; Willie et al., 2018). The study was approved by the University Hospitals of Cleveland Institutional Review Board (#09-13-01), the Division of Microbiology and Infectious Diseases/NIAID/National Institutes of Health (NIH) (#13-0067), and the Madagascar Ministry of Health Ethics Committee (#099). Written informed consent was obtained from all subjects, or subject guardians, prior to enrollment.

The Malagasy population sampled included rural communities in the Mandoto district (*n* = 167) and a migrant population in the rural Ampasimpotsy community of Tsiroanomandidy district (*n* = 44) relocated from the capital city, Antananarivo. These communities are located in the highlands fringe region and self-identified as being predominantly of the Merina highland ethnic group (Howes et al., 2017). These populations were considered genetically representative of Malagasy populations targeted for PQ therapy.

### Sample Collection and Processing

Samples were collected between March and August 2015 from apparently healthy individuals of all ages during a study screening for asymptomatic malaria infections and G6PD status. Most individuals were male (92%) due to the parallel objective of qualitative phenotypic G6PD characterization. Details of the communities surveyed have been previously documented (Howes et al., 2017). Two hundred μL of capillary blood was collected into K^+^-EDTA microtainers for DNA extraction and subsequent *CYP2D6* genotyping. DNA was extracted from 50 to 100 μL of each sample using a QIAamp® DNA Micro Kit (QIAGEN, Germantown, MD). The remaining blood sample and extracted DNA were stored at −20°C.

### *CYP2D6* Genotype Analysis and Translation Into Phenotype

Samples were genotyped using commercially available TaqMan™ genotyping assays on a custom-designed OpenArray (Thermo Fisher Scientific, Waltham, MA), as recommended by the manufacturer on a QuantStudio™ 12K Flex Real-Time PCR System (Thermo Fisher Scientific, Waltham, MA). Data were analyzed with the TaqMan™ Genotyper Software and manually inspected. A total of 29 SNPs were tested, which allowed the identification of the presence of the following allelic variants using star allele nomenclature per PharmVar: CYP2D6*2, *3, *4, *6, *7, *8, *9, *10, *11, *12, *14, *15, *17, *29, *31, *35, *40, *41, *42, *44, *45, *49, *56, *59, *99, *100, and *101. CYP2D6*1 was assigned if no variants were identified. In addition, all samples were interrogated for the presence of CNVs including the CYP2D6*5 gene deletion, duplications, hybrid genes, and tandem arrangements utilizing a previously published quantitative multiplex method that interrogates four gene regions (Gaedigk et al., 2012). Long-range PCR reactions were performed to confirm and further characterize CNV results following published protocols (Gaedigk et al., 2007; Gaedigk et al., 2010; Gaedigk et al., 2019). Additional information is provided in Supplementary Table S1 using the PharmVar recommended template for reporting genotyping method details.

Star alleles were called using nomenclature detailed by PharmVar. Subsequently, activity scores (AS) were determined for each diplotype and translated into phenotype as recommended by CPIC (Caudle et al., 2020). Briefly, values of 0, 0.25, 0.5, or 1 were assigned to each allele and the sum of both values added to determine a diplotype’s AS. Alleles with gene duplications received a value double that of its single counterpart. Values assigned to each allele are provided in Table 1. AS groups were translated into phenotype as follows: UM, AS > 2.25; NM, AS = 1.25, 1.5, 2.0, and 2.25; IM, AS = 0.25, 0.5, 0.75, and 1.0; PM, AS = 0.

**TABLE 1:** *CYP2D6* allele frequencies in the Madagascar study population^‡^.

**Alleles found in this study** ^a^	**Number of alleles**	**Allele frequency**	**95% CI** ^b^	**Value for activity score calculation** ^c^
*1	151	35.78	31.12, 40.45	1.0
*1x2	1	0.24	0, 0.71	2.0
*2	27	6.40	4.02, 8.78	1.0
*2x2	2	0.47	0, 1.14	2.0
*4	9	2.13	0.73, 3.54	0
*4x2	6	1.42	0.27, 2.57	0
*5	7	1.66	0.42, 2.90	0
*10	72	17.06	13.40, 20.72	0.25
*17	46	10.90	7.87, 13.93	0.5
*29	28	6.64	4.21, 9.06	0.5
*35x2	1	0.24	0, 0.71	2.0
*36+*10	51	12.09	8.91, 15.26	0.25
*36x2+*10	1	0.24	0, 0.71	0.25
*40	1	0.24	0, 0.71	0
*41	15	3.55	1.75, 5.36	0.5
*45	3	0.71	0, 1.53	1.0
*100	1	0.24	0, 0.71	0

^‡^ Number of subjects = 211.

^a^ Alleles *3, *6, *7, *8, *9, *11, *12, *14, *15, *31, *42, *44, *49, *56, *59, *99, and *101 were tested but were not found in this study.

^b^ 95% Confidence interval values represent lower limit and upper limit.

^c^ Value for Activity Score calculation as recommended by the CYP2D6 Diplotype-Phenotype table at https://www.pharmgkb.org/page/cyp2d6RefMaterials.

### Statistical Analysis

95% Confidence interval (95% CI) lower and upper limit values for the allele frequencies were calculated as described in http://awarnach.mathstat.dal.ca/∼joeb/biol3046/PDFs/PopGen1_HWE.pdf. Expected genotype numbers were calculated, and chi-square (χ^2^) deviation between the observed and expected numbers was calculated for each genotype. A χ^2^ value greater than 3.841 at one degree of freedom (d.f.) and *α* = 0.05 is considered significant (*p* < 0.05). The online calculator https://www.socscistatistics.com/pvalues/chidistribution.aspx was used to generate a *p*-value from the cumulative χ^2^ score.

## Results

### *CYP2D6* Allele Frequencies

All alleles detected were counted and calculated for their respective frequencies (Table 1). Nine alleles were detected (CYP2D6*2, *4, *10, *17, *29, *40, *41, *45, and *100). In addition, we detected CYP2D6*5 gene deletions, gene duplications (CYP2D6*1x2, *2x2, *4x2, and *35x2), and alleles with tandem arrangements (CYP2D6*36+*10 and *36x2+*10). The most common nonfunctional allele was CYP2D6*4 (2.13%), followed by *5 (1.66%) and the *4x2 gene duplication (1.42%). Among the decreased function alleles, those with an AS of 0.25 (CYP2D6*10, 17.06%; *36+*10, 12.09%) were more common compared to those with an AS of 0.5 (CYP2D6*17, 10.90%; *29, 6.64%; *41, 3.55%). Alleles found in the Malagasy population and their frequencies are summarized in Table 1.

### *CYP2D6* Genotype Frequencies and Predicted Phenotypes

Forty-one genotypes were inferred, with frequencies ranging from 0.47% (1/211) to 14.22% (30/211) (Table 2). Six genotypes (CYP2D6*1/*1, *1/*2, *1/*10, *1/*17, *1/*29, and *1/*36+*10) were present at frequencies >5%, with CYP2D6*1/*10 being the most common (14.22%). Among the 41 observed genotypes, two (4.88%) were predicted to be UM (AS, 3.0), 18 (43.9%) NM (AS range, 1.25–2.25), and 21 (51.22%) IM (AS range, 0.25–1.0). No PM subjects were identified. Based on the low frequency of nonfunctional alleles observed in our population sample (24 of 422 alleles, or 5.67%), about 0.32% of the population would be expected to be PMs.

**TABLE 2:** *CYP2D6* genotype frequencies and predicted phenotypes in the Madagascar study population^§^.

**Genotypes found in this study**	**Number of subjects**	**Genotype frequency**	**Activity score** ^a^	**Predicted phenotype**
*1/*2x2	1	0.47	3.0	UM
*1/*35x2	1	0.47	3.0	UM
*1/*10	30	14.22	1.25	NM
*1/*36+*10	19	9.00	1.25	NM
*1/*36x2+*10	1	0.47	1.25	NM
*2/*10	4	1.90	1.25	NM
*2/*36+*10	2	0.95	1.25	NM
*36+*10/*45	1	0.47	1.25	NM
*1/*17	21	9.95	1.5	NM
*1/*29	12	5.69	1.5	NM
*1/*41	4	1.90	1.5	NM
*2/*17	3	1.42	1.5	NM
*2/*29	3	1.42	1.5	NM
*2/*41	1	0.47	1.5	NM
*1/*1	18	8.53	2.0	NM
*1/*2	13	6.16	2.0	NM
*1/*45	1	0.47	2.0	NM
*2/*45	1	0.47	2.0	NM
*2x2/*5	1	0.47	2.0	NM
*1x2/*10	1	0.47	2.25	NM
*4/*36+*10	2	0.95	0.25	IM
*4x2/*36+*10	1	0.47	0.25	IM
*5/*10	3	1.42	0.25	IM
*10/*40	1	0.47	0.25	IM
*36+*10/*100	1	0.47	0.25	IM
*4/*29	2	0.95	0.5	IM
*4x2/*41	1	0.47	0.5	IM
*10/*10	7	3.32	0.5	IM
*10/*36+*10	6	2.84	0.5	IM
*36+*10/*36+*10	3	1.42	0.5	IM
*10/*17	5	2.37	0.75	IM
*10/*29	6	2.84	0.75	IM
*10/*41	2	0.95	0.75	IM
*17/*36+*10	7	3.32	0.75	IM
*36+*10/*41	6	2.84	0.75	IM
*1/*4	5	2.37	1.0	IM
*1/*4x2	4	1.90	1.0	IM
*1/*5	3	1.42	1.0	IM
*17/*17	2	0.95	1.0	IM
*17/*29	5	2.37	1.0	IM
*17/*41	1	0.47	1.0	IM

^§^ Number of subjects = 211.

^a^ Activity Score was calculated as recommended by CPIC (Caudle et al., 2020) using the CYP2D6-specific information table available at https://www.pharmgkb.org/page/cyp2d6RefMaterials. UM, ultrarapid metabolizer; NM, normal metabolizer; IM, intermediate metabolizer.

All six genotypes with frequencies >5% were predicted to be NM, and they were distributed equally among three AS groups [1.25, 1.5, and 2.0 (*n* = 2 each)]. Among the genotypes predicted to be NM, those with an AS of 1.25 were more common [six genotypes among 57 samples (27%)] compared to those with an AS of 1.5 [six genotypes among 44 samples (20.85%)] or 2.0 [five genotypes among 34 samples (16.11%)]. The distribution of the genotypes, predicted to be IM, according to their AS was as follows: AS 0.25, five genotypes among eight samples (3.79%); AS 0.5, five genotypes among 19 samples (9.01%); AS 0.75, five genotypes among 26 samples (12.32%); and AS 1.0, six genotypes among 20 samples (9.48%). Among those with an AS of 0.25 and 0.5, the nonfunctional CYP2D6*4, *4x2, and *5 alleles were present in a total of nine samples (33.33%). All genotypes, their frequencies, and translation via AS into phenotype are summarized in Table 2.

### Chi-Square Statistic

A χ^2^ test-based comparison between the observed and expected genotype numbers for all 41 genotypes (Supplementary Table S2) showed that the total expected genotype number (*n* = 189) was significantly lower than the total observed genotype number (*n* = 211) [cumulative χ^2^ = 84.39, 23 d.f. (41−18; 1 d.f. for having a finite sample, and 17 d.f. for the total number of alleles), *p* < 0.00001]. The observed and expected genotype numbers did not differ from each other for 36 genotypes including the six genotypes with frequencies >5%. Five genotypes, four observed in one subject each, contributed to the overall deficit in the expected genotype number. These five genotypes are highlighted in Supplementary Table S2.

## Discussion

*CYP2D6* is one of the Tier 1 Very Important Pharmacogenes with extensive variation (https://www.pharmgkb.org/vip/PA166170264/overview). *CYP2D6* allele frequencies differ considerably among ethnically distinct populations (Gaedigk et al., 2017). A comprehensive summary of allele frequencies across populations including source citations can be found on the PharmGKB website (https://www.pharmgkb.org/page/cyp2d6RefMaterials). Based on the latter, CYP2D6*4 has the highest frequency of all nonfunctional alleles in the European group (15–20%; elsewhere, 5–12%), whereas CYP2D6*5, observed at frequencies of 3–6% in most populations, occurs more frequently in South African populations. The CYP2D6*10 decreased function allele is the most common variant allele in the East Asian group (frequencies 9–64%; elsewhere, 2–8%), whereas CYP2D6*17 and *29 are more prominent in the Sub-Saharan African and African American/Afro-Caribbean groups, as compared to others (12–20% and 6–12%, respectively; elsewhere, 1–3% and 1–2%, respectively). CYP2D6*41 allele frequency is higher in Middle Eastern populations than in other ethnic groups (14–18%; elsewhere, 1–10%). Some alleles (e.g., CYP2D6*2, *4, or *5) are found at variable frequencies in almost every population studied, whereas others have only been found in a few populations to date (e.g., CYP2D6*100 and *101 in Trinidadians of Indian ancestry). Finally, populations including South African, Caribbean, and others with diverse founding populations and admixture often reveal unique allele frequency patterns (Nofziger et al., 2020).

In the Malagasy population, although some allele frequencies were comparable to those found in other populations, allele and genotype frequency distribution patterns were rather unique. This is particularly true for CYP2D6*10, *17, and *29. The CYP2D6*10 allele frequency (17.06%) is considerable higher compared to Europeans or Africans, although not quite as high as those observed across East Asia. In contrast, CYP2D6*17 and *29 allele frequencies (10.90 and 6.64%, respectively) are more similar to those in African than in other populations, albeit not as high as in some African populations. Of note was the high frequency (12.09%) of the CYP2D6*36+*10 tandem rearrangement, which is typically only seen in East Asians (16–30%). These predominantly East Asian or African alleles were observed to come together as CYP2D6*10/*17 (2.37%), CYP2D6*10/*29 (2.84%), and CYP2D6*17/*36+*10 (3.32%) genotypes. Finding these allelic variants at notable frequencies is in full agreement with the archaeological and other genomic evidence that Malagasy populations originate from Asian and African populations and constitute a unique, heavily admixed population. Furthermore, the discovery of a subject carrying the rare CYP2D6*100 allele underscores the need to thoroughly characterize populations with a high level of admixture.

Vivax malaria relapse following PQ treatment has predominantly been observed in patients carrying decreased function alleles such as CYP2D6*10, *36, and *41, and nonfunctional alleles such as CYP2D6*4 and *5 in various combinations giving rise to IM and PM phenotypes (Bennett et al., 2013; Ingram et al., 2014; Baird et al., 2018; Dijanic et al., 2018; He et al., 2019; Martin Ramirez et al., 2020; Mat Salleh et al., 2020; Silvino et al., 2020). In most cases where there was drug failure, the following genotypes were observed: CYP2D6*4/*41 (Bennett et al., 2013), *5/*41 (Ingram et al., 2014), *5/*10, *10/*10, and *10/*41 (Baird et al., 2018; Mat Salleh et al., 2020), *1/*4 (Martin Ramirez et al., 2020), and *2/*36 (He et al., 2019), all predicting IM status. In addition, there were two genotypes, CYP2D6*5/*6 (Bennett et al., 2013) and *4/*5 (Baird et al., 2018; Dijanic et al., 2018) that were reported to completely abolish enzyme activity and give rise to PM status. Interestingly, some studies have shown that relapses also occurred in patients with CYP2D6*1/*10 and *2/*10 (Baird et al., 2018) genotypes, predicting NM status. However, subjects in these studies (Bennett et al., 2013; Ingram et al., 2014; Baird et al., 2018; Dijanic et al., 2018; He et al., 2019; Martin Ramirez et al., 2020; Mat Salleh et al., 2020; Silvino et al., 2020) may harbor rare or novel alleles that may not have been detected by screening for the common allelic variants only. Similar results have been reported when the AS system (activity value >1.0, normal function; activity value ≤1.0, decreased function), instead of genotypes and/or predicted phenotypes, was considered to determine the odds or risk of relapse; AS ≤ 1.0 were significantly associated with higher odds or risk of relapse (Baird et al., 2018; Brasil et al., 2018; Silvino et al., 2020). In this current Malagasy sample set, the decreased function alleles were highly prevalent at 50.48%, whereas nonfunctional alleles were noticeably less common at 5.67%. Given these frequencies, 34.6% of the individuals are predicted to be IM with an AS ≤ 1.0, whereas PMs are likely rare at <1%. Therefore, the Malagasy population presents a unique opportunity to investigate PQ metabolism and therapeutic efficacy more systematically with respect to *CYP2D6* genotypes. This is especially relevant considering that a new policy to start administration of PQ may soon be executed Madagascar NMCP (National Malaria Control Programme of Madagascar, 2017).

Given the importance of CYP2D6-mediated metabolism for PQ efficacy, the precise role that this enzyme plays in PQ metabolism and pharmacokinetics is now being studied *in vivo* (Tekwani et al., 2015; Avula et al., 2018). Although still limited, there is recent information on the consequences associated with differential CYP2D6 metabolism status of PQ (Goncalves et al., 2017; Ariffin et al., 2019; Spring et al., 2019). A pharmacokinetic study conducted in 14-year old African children showed that PM children (AS = 0.0) had higher levels of PQ, as compared to IM (AS = 0.5, 1.0), NM (AS = 1.5, 2.0), and UM (AS = 3.0) children, strongly suggesting compromised CYP2D6-mediated PQ metabolism (Goncalves et al., 2017). Another study investigated the effect of the 100C>T SNP on PQ metabolism in healthy volunteers from Malaysia and showed that individuals being heterozygous or homozygous for the T allele had less PQ biotransformed into an active metabolite (Ariffin et al., 2019). However, one limitation of this study was that only one SNP (100C>T) was tested as a key marker for CYP2D6*10. As this SNP is also present on a number of other alleles, including the nonfunctional CYP2D6*4 allele (reported to occur at ∼3% frequency in Malay populations, https://www.pharmgkb.org/page/cyp2d6RefMaterials), the CYP2D6*10 frequency is likely over-estimated, and the contribution of other allelic variants that have been reported in Malay populations (Teh et al., 2001; Chin et al., 2016; Goh et al., 2017) was not accounted for. A more comprehensive study assessed the impact of CYP2D6 metabolizer status on the plasma levels of suspected active phenolic metabolites of PQ (Spring et al., 2019). In this United States-based pharmacokinetic study, volunteers were characterized as genotypic NM (AS = 1.5, 2.0), IM (AS = 0.25, 0.5, 1.0), and PM (AS = 0.0). Consistent with the body of literature, data further corroborated that PQ metabolism was decreased in IM and PM individuals when compared to NM individuals (Spring et al., 2019). Collectively, these findings (Goncalves et al., 2017; Ariffin et al., 2019; Spring et al., 2019) provide an encouraging basis for performing studies in vivax-endemic areas, such as Madagascar, to further unravel the pathways of PQ metabolism and characterize the impact of variable CYP2D6 activity on *P. vivax* treatment outcomes. Furthermore, by comparing PQ metabolite profiles of subjects with different genotypes, one may be able to assess which *CYP2D6* allelic variants impact the production of oxidative metabolites posited to be necessary for radical cure of *P. vivax* infection.

Being a unique population, Madagascar provides an opportunity to gain new insights into PQ metabolism and efficacy. First, in the present study, we tested for a discrete set of *CYP2D6* alleles, selected out of over 130 alleles that have been defined to date (https://www.pharmgkb.org/page/cyp2d6RefMaterials). It is likely that some of the individuals in our study population may have undetected novel or rare alleles, including structural variants, which may or may not be functionally relevant. This limitation may influence the observed frequency of *CYP2D6* alleles, especially the normal function CYP2D6*1 allele, which is assigned if no other SNPs are identified. Other alleles may default to CYP2D6*2, or other, however, depending whether they are on a CYP2D6*1-like or *2-like backbone. We refer to Nofziger et al. (Nofziger et al., 2020) for additional information and example of alleles defaulting to CYP2D6*10 assignment. Clearly, sequencing-based approaches will better identify novel or rare alleles than genotype panels. Second, variation in other drug-metabolizing enzymes, drug transporters and/or drug targets may contribute to PQ metabolism and response, and contribute overall to therapeutic efficacy (Li et al., 2003; Silvino et al., 2016; Sortica et al., 2017; Ariffin et al., 2019; Chamnanphon et al., 2020). An early *in vitro* study identified hepatic CYP1A2 and CYP2D6 as those responsible for the metabolism of PQ (Li et al., 2003). Other reports indicate that polymorphically expressed monoamine oxidase A (Ariffin et al., 2019), CYP2C8 (Silvino et al., 2016), CYP2C19 (Ariffin et al., 2019; Chamnanphon et al., 2020), UDP-glucuronosyltransferase 2B7 (Chamnanphon et al., 2020), ATP-binding cassette transporter G2 (Chamnanphon et al., 2020), and solute carrier organic anion transporters 1A2, 1B1, and 2B1 (Sortica et al., 2017) may also contribute to PQ metabolism, and thus play a role in variable treatment outcomes for vivax malaria. In addition, in a recent genome-wide association study, two signals (rs62103056 on chromosome 18 and a 30-kb intergenic region on chromosome 12) were significantly associated with tafenoquine efficacy in patients with *P. vivax* malaria (St Jean et al., 2020). Biological relevance and replication of these findings are, however, needed. It is therefore possible that additional variation in *CYP2D6* and/or potential “modifiers” elsewhere in the genome may affect PQ metabolism. Untangling not only the complex routes of PQ metabolism, but also the contribution of genomic variation on metabolite formation, especially those that are active against *P. vivax* stages, are essential to eventually develop optimized PQ dosing strategies. Finally, another open question is substrate-specific allele function (Marcath et al., 2019). In other words, it is unknown whether, and if so to what extent, PQ is metabolized in a substrate-specific manner especially by alleles categorized as decreased function. This concern is highly relevant given the results of an *in vitro* study conducted by Saito et al. (Saito et al., 2018). In this study, PQ was used as a substrate and 5-hydroxylation was used as a measure of CYP2D6 activity. CYP2D6*17 retained 42% of activity compared to the CYP2D6*1 reference, confirming the decreased function status of this allelic variant (and value of 0.5 used to calculate AS). There was no 5-hydroxylation activity detected for CYP2D6*36 and several other nonfunctional alleles, confirming their no function status (and value of 0 used for AS calculation). Interestingly, no 5-hydroxylation activity was detected for CYP2D6*10 and CYP2D6*29. The absence of detectable *in vitro* activity for these alleles is especially concerning, given that both are relatively common in many populations plagued by malaria, including the Malagasy. Thus, future studies need to address whether the CPIC recommended method of translating AS into phenotype is appropriate to predict CYP2D6 activity for PQ treatment.

In conclusion, although allele and genotype frequencies need to be validated in a larger population sample, this snapshot study is highly informative as it revealed the presence of numerous decreased function alleles and predicts that a substantial proportion of this population has decreased CYP2D6 activity. In addition, this study provides new insight regarding the genetic admixture of *CYP2D6* in a portion of the Malagasy population. Consistent with the historical peopling of Madagascar, the alleles associated with African and East Asian/Indonesian populations are among those most highly prevalent. Finally, current knowledge provides fresh insights about *P. vivax* elimination challenges for the global population at risk, and enables future studies to address those challenges with the goal to optimize PQ treatment across human genetic diversity. Such efforts would not only benefit successful *P. vivax* treatment, and eventual elimination, in Madagascar but all populations afflicted by this parasite.

## Data Availability

The original contributions presented in the study are included in the article/Supplementary Material, further inquiries can be directed to the corresponding authors.
